# The Endocannabinoid System as a Therapeutic Target in Glaucoma

**DOI:** 10.1155/2016/9364091

**Published:** 2016-01-12

**Authors:** Elizabeth A. Cairns, William H. Baldridge, Melanie E. M. Kelly

**Affiliations:** ^1^Department of Pharmacology, Dalhousie University, Halifax, NS, Canada B3H 4R2; ^2^Department of Medical Neuroscience, Dalhousie University, Halifax, NS, Canada B3H 4R2; ^3^Department of Ophthalmology and Visual Sciences, Dalhousie University, Halifax, NS, Canada B3H 4R2

## Abstract

Glaucoma is an irreversible blinding eye disease which produces progressive retinal ganglion cell (RGC) loss. Intraocular pressure (IOP) is currently the only modifiable risk factor, and lowering IOP results in reduced risk of progression of the disorder. The endocannabinoid system (ECS) has attracted considerable attention as a potential target for the treatment of glaucoma, largely due to the observed IOP lowering effects seen after administration of exogenous cannabinoids. However, recent evidence has suggested that modulation of the ECS may also be neuroprotective. This paper will review the use of cannabinoids in glaucoma, presenting pertinent information regarding the pathophysiology of glaucoma and how alterations in cannabinoid signalling may contribute to glaucoma pathology. Additionally, the mechanisms and potential for the use of cannabinoids and other novel agents that target the endocannabinoid system in the treatment of glaucoma will be discussed.

## 1. Introduction

The endocannabinoid system (ECS) including cannabinoid receptors, cognate biosynthetic and degradative enzymes, and endocannabinoids, such as anandamide (AEA) and 2-arachidonoylglycerol (2-AG), is present in both anterior and posterior ocular tissues including the retina (reviewed in [1]). The presence of these components supports an important role for the ocular ECS in the endogenous signalling of both the anterior and posterior eye. Consistent with this, application of cannabinoids to the eye produces a variety of effects, notably hyperemia, reduced tear production, and a reduction in intraocular pressure (IOP) [2] (reviewed in [1, 3]). Of these effects, the IOP lowering properties of cannabinoids have attracted considerable attention with respect to the possibility of developing cannabinoid-based therapeutics for glaucoma [1, 3–6], a progressive irreversible blinding eye disease, which is the second leading cause of blindness worldwide [7].

Glaucoma represents a group of optic neuropathies characterized by cupping of the optic nerve head and selective retinal ganglion cell (RGC) loss. While IOP is a major modifiable risk factor, the exact relation between IOP and RGC death is not completely clear. Patients may have glaucoma without having elevated IOP or conversely have elevated IOP but not have glaucoma [7, 8]. However, regardless of the initial IOP, for every mmHg reduction in IOP, there is a 10% reduced risk of progression of the disorder [9]. While cannabinoids were initially exploited in glaucoma solely based on their IOP lowering properties [2], recent evidence suggests that modulation of the ECS may also be neuroprotective (reviewed in [1]).

This review will discuss the use of cannabinoids in glaucoma, presenting pertinent information regarding the pathophysiology of glaucoma and how alterations in cannabinoid signalling may contribute to glaucoma pathology. Additionally, the mechanisms and potential of cannabinoids as ocular hypotensive agents and neuroprotectants in the treatment of glaucoma will be discussed.

## 2. The Endocannabinoid System in the Eye

The endocannabinoid system is present throughout most ocular tissues, including anterior eye tissues responsible for the generation of IOP (as outlined below), and in the retina (reviewed in [1]). The endocannabinoids 2-AG and AEA are found throughout the eye, with the exception of the lens [5, 10]. 2-AG and AEA both bind to cannabinoid 1 receptor (CB_1_) and cannabinoid 2 receptor (CB_2_). CB_1_ is expressed in the ciliary body, trabecular meshwork, Schlemm's canal, and retina [11–19]. With respect to CB_2_, Cécyre and colleagues [20] reported that electroretinographic responses were altered in CB_2_ knockout mice, indicating that CB_2_ is present in the retina and may contribute to normal visual function. However, the localization of CB_2_ expression has been quite controversial. CB_2_ mRNA has been reported in the retina [16], but the lack of good immunohistochemical markers has hampered studies attempting to study the expression pattern of the receptor. In 2011, López and colleagues [19] reported immunoreactive staining in the retinal pigmented epithelium and much of the inner retina; however, a recent study in a small number of nonhuman primates found that CB_2_ immunoreactivity was localized only to Müller cells [21] (also reported in* in vitro* data from primary cultures and retinal explants [22]). Additionally, pharmacological studies have suggested that CB_2_ may also be expressed in the anterior eye [23].

Several noncannabinoid receptor targets of endocannabinoids have also been localized to the eye. Transient receptor potential type vanilloid 1 receptor (TRPV1), a target of AEA, is expressed in the retina, including RGCs and retinal microglia [24]. GPR18 is a cannabinoid-related receptor that is activated by* N*-arachidonoyl glycine (NAGly), a metabolite of AEA [25]. GPR18 has been localized to the cornea, ciliary epithelium, iris, and retina [26, 27]. Investigation of the expression patterns of other cannabinoid-related receptors, such as GPR55 and GPR119, has been limited to date, although there is some evidence which supports the expression of GPR55 in the anterior eye and in rods in the retina [28, 29].

**Figure 1:**
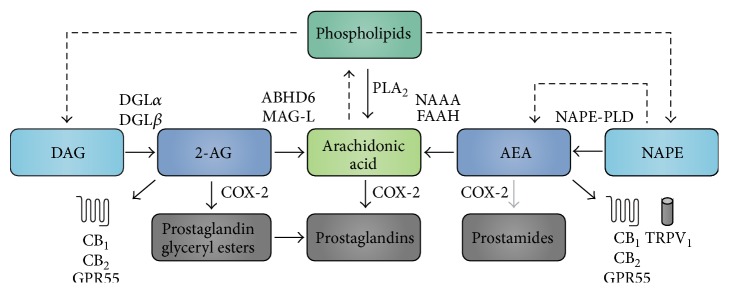
Overview of AEA and 2-AG production and metabolism. The endocannabinoids 2-AG and AEA are formed from arachidonic acid-containing phospholipids. 2-AG is formed from DAG by DGL*α* or DGL*β* and is metabolized either via COX-2 to form prostaglandin glyceryl esters or by ABHD6 or MAG-L to form arachidonic acid. The production of AEA occurs through conversion of NAPE by either a NAPE-PLD dependent or independent pathway. Once formed, AEA is broken down by either NAAA or FAAH to form arachidonic acid or occasionally by COX-2 to form prostamides. Arachidonic acid can be synthesized via phospholipase A_2_ (PLA_2_) from phospholipids and is also broken down by COX-2, forming prostaglandins and other eicosanoids. Additionally, arachidonic acid can be converted back to a phospholipid [30, 31]. Dashed lines indicate multistep pathway; gray lines indicate weak pathway.

Endocannabinoid signalling is determined by the balance between production and degradation (Figure 1). Endocannabinoids are derived on demand from arachidonic acid-containing phospholipids after hydrolysis by phospholipases [30, 31]. Studies localizing the enzymes catalyzing the production of AEA and 2-AG in the eye have been minimal to date. However, two enzymes involved in the synthesis of 2-AG, diacylglycerol lipase-*α* and lipase-*β* (DGL*α* and DGL*β*), have been localized to ocular structures in mouse [32]. As additional antibodies become available, it is more than likely that other enzymes will be localized to these tissues as well. AEA is primarily metabolized by the enzyme fatty acid amide hydrolase (FAAH), and also by* N*-acylethanolamine-hydrolyzing acid amidase (NAAA) [30, 31, 33]. 2-AG is metabolized by both monoacylglycerol lipase (MAG-L) and *α*,*β*-hydrolase domain-containing 6 (ABHD6) [31, 34–36]. In addition to metabolizing arachidonic acid, evidence suggests that cyclooxygenase-2 (COX-2) can also directly metabolize both AEA and 2-AG to prostaglandin-ethanolamides (prostamides) and prostaglandin glyceryl esters, respectively [37, 38]. In fact, the affinity for and efficacy of the metabolism of 2-AG by COX-2 are comparable to those of arachidonic acid [39]. The nonselectivity of COX-2 between arachidonic acid and 2-AG highlights the interconnectedness of the ECS and the eicosanoid system [38]. FAAH and COX-2 immunoreactivity was found in the ciliary body, and in some retinal cells, including RGCs [15, 32, 40, 41]. A similar pattern of immunoreactivity has been found for MAG-L and ABHD6 in the mouse retina [32]; however, so far only one study has reported the presence of MAG-L in porcine trabecular meshwork tissues, though this has yet to be confirmed with immunostaining [41]. NAAA was localized only to mouse retinal pigment epithelium and has yet to be investigated in the anterior eye [32].

## 3. Alterations in ECS Signalling in Glaucoma and Retinal Disease 

Several studies have demonstrated fluctuations in endocannabinoid tone during disease states (reviewed in [42]). Deviations from homeostasis, including injury, inflammation, or even acute changes, will usually result in the elevation of at least one endocannabinoid in the tissues involved. This change may serve to help reestablish normal physiologic levels of other endogenous mediators and activate pathways to help protect the cells from death (reviewed in [42, 43]). Nevertheless, chronic perturbation of the endocannabinoid system is not always protective and may under certain circumstances contribute to the pathology. Various disease states involve either up- or downregulation of endogenous cannabinoids, including many neurological disorders such as Parkinson's disease (increased AEA and decreased 2-AG), Alzheimer's disease (increased 2-AG), amyotrophic lateral sclerosis (increased AEA and 2-AG), multiple sclerosis (decreased AEA and 2-AG), and neuropathic pain (increased AEA and 2-AG) (reviewed in [42, 43]).

Investigation of pathology-induced changes in the endocannabinoid system in the eyes of both human and animal models has so far been limited to relatively few studies (for review, see [1]). In diabetic retinopathy, 2-AG was increased in the iris, and AEA increased in the retina, ciliary body, and cornea. Similarly, AEA was increased in age-related macular degeneration in the retina, choroid, ciliary body, and cornea [10]. To date, only one study has looked at endocannabinoid levels in human glaucomatous eyes. Chen and colleagues [5] found that 2-AG and PEA (*N*-palmitylethanolamide, an AEA analogue) were reduced in ciliary body, and PEA was also reduced in the choroid in postmortem eyes from glaucoma patients. Interestingly, there was no significant difference in AEA levels in any of the ocular tissues measured [5]; however, retinal AEA was found to be decreased 6 hours after reperfusion in a rat model of transient high IOP-induced ischemia, and this decrease in AEA was associated with increased FAAH activity [44].

Maihöfner and colleagues [40] found that COX-2 expression was significantly reduced in nonpigmented ciliary epithelial cells of human eyes with primary open-angle glaucoma. Furthermore, decreased endocannabinoid metabolites were found in sampled aqueous humor in these eyes [40]. This suggests that, under certain glaucomatous conditions, endocannabinoid metabolism might shift towards non-COX-2-dependent mechanisms, perhaps a reflection of increased FAAH activity as suggested by Nucci et al. [44]. However, in a model of transient retinal ischemia in rats, COX-2 expression was found to be increased in the retina and was associated with neurodegeneration [45]. Further work is warranted in order to clarify the relationship between COX-2 and endocannabinoid signalling in glaucoma pathology.

A study by Nucci and colleagues [44] also found that CB_1_ and TRPV1 expression was reduced in the retinas of a rat model of ocular hypertension. Data from another study were contradictory; in the DBA2J mouse model of glaucoma, TRPV1 immunoreactivity appeared to be increased, particularly in the inner retina. Though it is possible that this is a reflection of shift in the expression of TRPV1 from the soma to dendrites, these results may demonstrate that changes in expression of TRPV1 in the retina may be isolated to specific cell types, making changes difficult to detect when analyzing whole retinal expression [24].

Taken together, the data indicate that changes in the ECS occurred in ocular pathology including glaucoma, suggesting that it may be possible to improve glaucomatous outcomes through therapeutic modification of the endocannabinoid system. For example, this may include strategies directed at regulation of IOP and/or increased RGC survival by restoring endocannabinoids to their nonpathological levels or by directly activating cannabinoid receptors and downstream signalling molecules to compensate for these changes.

## 4. Cannabinoid Modulation of IOP

All current pharmacotherapies for glaucoma target IOP [46–48]. Cannabinoids modulate IOP, a phenomenon repeatedly demonstrated in studies using administration of endogenous or exogenous cannabinoids in rodents, rabbits, and nonhuman primates, and this ocular hypotensive action of cannabinoids has been a focus of studies of the therapeutic use of cannabinoids in the management of glaucoma. A few small human studies have reported efficacy of the phytocannabinoid Δ^9^-tetrahydrocannabinol (Δ^9^-THC) and the synthetic CB_1_ agonist WIN 55,212-2 in decreasing IOP [49–51]. Merritt et al. [52] found that topical application of Δ^9^-THC significantly decreased IOP in patients with glaucoma and did not elicit psychotropic effects noted in previous studies using smoked marijuana [53]. Another small study showed successful IOP reduction with topical application of the CB_1_ agonist WIN 55,212-2 in patients with glaucoma who were resistant to conventional treatment [49]. In general, the hypotensive effects of cannabinoids on IOP are largely due to actions at CB_1_ [4, 49, 54–56]. However, these actions on IOP may also involve CB_1_-independent effects [4, 26, 49, 54–58].

**Figure 2:**
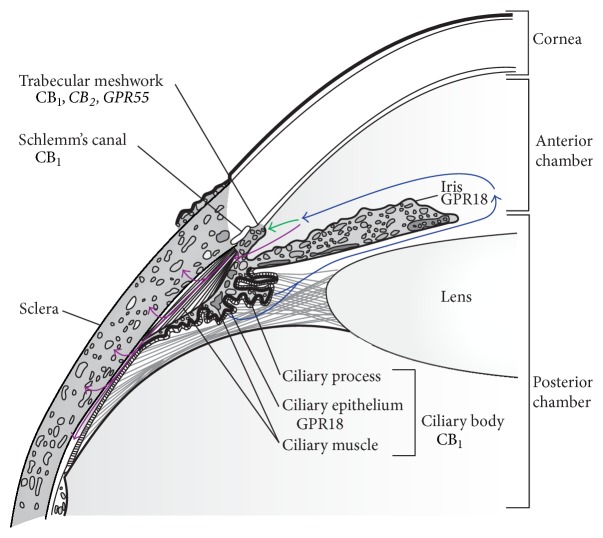
Cannabinoid-mediated alterations of the production and filtration of aqueous humor. Aqueous humor is formed by secretions from the ciliary body. Circulating aqueous humor (blue), flowing from the ciliary body in the posterior chamber to the anterior chamber, is filtered out of the eye through two different outflow pathways: the trabecular meshwork pathway (green) and the uveoscleral pathway (purple). The trabecular meshwork pathway involves the flow of aqueous humor through the trabecular meshwork to Schlemm's canal, where it will exit through the episcleral veins. The uveoscleral pathway involves the flow of aqueous humor from the iridocorneal angle to the posterior chamber through the ciliary body, and out through the supraciliary and suprachoroidal spaces. CB_1_, the major contributor to the IOP lowering effects of Δ^9^-THC and WIN 55,212-2, has been localized to the ciliary body, trabecular meshwork, and Schlemm's canal [11, 13–15, 17]. The IOP lowering effects of NAGly and Abn-CBD, and possibly CBG-DMH, are due to the activation of GPR18, which has been localized to the ciliary epithelium and iris [26, 58]. Additional pharmacological evidence has suggested that CB_2_ and GPR55 are localized within the trabecular meshwork [23, 28]; the contribution of these receptors to changes in IOP is unknown. COX-2 derived prostaglandins and prostamides are purported to exert actions through the uveoscleral pathway; however, the exact mechanism(s) is unclear [36, 62, 63]. Figure adapted from Riordan-Eva [94]. Italics indicate potential receptor localization which is not yet confirmed.

IOP is determined by the rate of aqueous humor production versus outflow. Aqueous humor is a transparent fluid produced by the eye that helps the eye keep its shape (important for proper optics) and acts as a modified circulatory system, removing wastes from avascular areas. Aqueous humor is produced by secretion from the ciliary body, which is located just beneath the iris (Figure 2), and is covered by a bilayered epithelium. Transporters on the ciliary epithelium allow the selective energy-dependent passage of certain solutes from the extracellular ciliary stroma into the posterior chamber [59]. CB_1_, GPR18, and various endocannabinoids have been localized to these tissues and may serve to modify secretion [5, 11, 13, 14, 17, 26]. Aqueous humor is filtered out of the eye through two different outflow pathways: the trabecular meshwork pathway and the uveoscleral pathway. The contribution of each of these pathways to the overall outflow is subject to variability between species (3–80%, depending on species, with significant interspecies variability) (reviewed in [60]).

The trabecular meshwork is located in the angle between the iris and cornea (Figure 2). Circulating aqueous humor will flow from the trabecular meshwork before being filtered through Schlemm's canal and exiting into the episcleral veins. Flow through this pathway may be altered by modifying resistance through the trabecular meshwork, modulated by the nearby ciliary muscle and through the smooth-muscle-like contractile properties of the trabecular meshwork itself [59]. CB_1_, CB_2_, and GPR55 receptors, as well as FAAH, have been localized at trabecular meshwork cells, indicating that they may also play a role in modulation at this site [23, 28, 41, 55, 61].

The uveoscleral pathway involves the flow of aqueous humor from the iridocorneal angle to the posterior chamber through the ciliary body. From here, the aqueous enters the supraciliary and suprachoroidal spaces [60]. Analogues of the endocannabinoid-derived prostamides used to target this pathway include the prostamide F_2*α*_ analogue bimatoprost. The exact mechanism by which these drugs act is unknown; however, it is thought that this may involve binding to a novel heterodimerized prostaglandin receptor (FP/altFP receptor), causing downstream remodeling of the ciliary muscle, and enlargement of this outflow route [36, 62, 63]. Therefore, COX-2 metabolism of endocannabinoids and modulation of the ECS in the uveoscleral system may play an important role in the treatment of glaucoma.

Although Δ^9^-THC and WIN 55,212-2 are known to activate both CB_1_ and CB_2_, their ocular hypotensive properties are primarily due to CB_1_ activation and do not involve CB_2_ [26, 57, 58]. For example, in control rats, Szczesniak et al. [58] found that the CB_2_ antagonist AM630 did not reduce the IOP lowering effect of WIN 55,212-2, while blocking the CB_1_ receptor using the cannabinoid AM251 abolished this effect. This finding was confirmed using CB_2_ knockout mice, in which there was no significant difference in IOP reduction after application of WIN 55,212-2 compared to control. Interestingly, application of WIN 55,212-2 in CB_1_ knockout mice produced an increase in IOP via unidentified non-CB_1_ actions [57].

The ocular hypotensive effect of CB_1_ appears to be mediated, at least in part, by *β*-adrenergic receptors (*β*ARs) [57]. Both *β*AR agonists and antagonists reduce IOP and have been used to lower IOP in glaucomatous patients: *β*AR antagonists, such as timolol, reduce aqueous humor secretion, while *β*AR agonists, such as isoproterenol, increase aqueous humor outflow by both trabecular and uveoscleral pathways. The CB_1_-mediated lowering of IOP is attenuated in *β*AR knockout mice, as are *β*AR agonist or antagonist effects in CB_1_ knockouts. Additionally, the catecholamine depleting agent reserpine was found to abolish the effects of both CB_1_ agonists and *β*AR antagonists, suggesting that CB_1_ activation may inhibit the release of norepinephrine [57]. The lack of direct actions of *β*AR agonists in CB_1_ knockout mice was suggested to occur via desensitization of *β*AR due to elevated noradrenergic tone. However, other interactions between CB1 and *β*AR, such as heterodimerization, which may result in alterations in *β*AR expression and pharmacology, cannot be ruled out [57].

Other ECS modulators appear to have IOP lowering effects that are independent of CB_1_ or CB_2_. Two behaviourally inactive cannabinoids, abnormal cannabidiol (Abn-CBD) and cannabigerol-dimethyl heptyl (CBG-DMH), lowered IOP in normotensive rats, and these effects were not blocked by coapplication of either CB_1_ or CB_2_ antagonists. The ocular hypotensive effect of Abn-CBD and CBG-DMH was, however, abolished by O-1918, an antagonist at GPR18 [58]. NAGly, an agonist at GPR18 [64], was also reported to lower IOP. The ocular hypotensive action of NAGly was mediated independently of CB_1_, CB_2_, and GPR55, as demonstrated through the use of knockout mice [26]. Collectively, these data support a role for GPR18 in IOP regulation. While the actions of NAGly did not involve GPR55, PEA was reported to increase aqueous humor outflow through the trabecular meshwork via activation of GPR55 and peroxisome proliferator-activated receptor *α* [28].

Taken together, this work suggests that the ECS plays a prominent role in aqueous humor dynamics and that drugs targeting both CB_1_ and non-CB_1_/CB_2_ receptors may be useful as ocular hypotensives. An increasing understanding of the localization of components of the ECS to tissues involved in aqueous humor production and outflow and the function of the ECS in regulation of IOP under normal and pathological conditions is still required.

## 5. Targeting the Endocannabinoid System for Novel Glaucoma Therapeutics

Many patients, despite the use of IOP lowering drugs, continue to show progressive vision loss. This suggests that strategies which target IOP as well as providing neuroprotection may be beneficial [65]. There is no current approved neuroprotective drug for glaucoma, and this is most likely a reflection of the lack of knowledge of the mechanisms leading to glaucomatous RGC loss [65]. However, a few components of this pathway have been determined. RGCs ultimately die by apoptosis caused by activation of proapoptotic pathways, namely, caspase activation, by excessive intracellular calcium [66]. The source of this intracellular calcium has yet to be determined; however, this more than likely arises from multiple sources [66]. Once this late-phase apoptosis is activated, neuroprotection is no longer possible as cellular function is irreparably compromised [66, 67]. Therefore, therapies which may reduce the calcium load on the cell may provide novel strategies for the treatment of glaucoma.

Many studies examining ECS modulation in models of glaucoma have found that, aside from IOP lowering effects, modulating the ECS is neuroprotective; this includes use of ligands acting directly at cannabinoid receptors as well as modulators of cannabinoid metabolism (summarized in Table 1) [24, 44, 45, 68–74]. Additionally, the neuroprotective properties of these compounds have been demonstrated in pressure-independent models of RGC loss, including neural excitotoxicity and axotomy [69, 71, 73], suggesting that ECS modulation may be neuroprotective in the retina independent of changes in IOP.

For example, Crandall and colleagues [68] found that, in a rat model of ocular hypertension, weekly administration of THC increased the number of surviving ganglion cells in both central and peripheral regions of affected retinas. Additionally, a recent study using topical administration of WIN 55,212-2 in a transient high IOP model indicated that significant cell loss was only seen in animals receiving vehicle and was not seen in cannabinoid-treated animals. This neuroprotective effect of WIN 55,212-2 was blocked with the CB_1_ antagonist AM251, and suggests that the increased RGC survival seen was due to the CB_1_ receptor activation [70].

Additional studies have confirmed these findings in other models. Hyperactivation of glutamate receptors has been used as a model of RGC death (with either NMDA or kainate), induced by intravitreal injection or direct bath application* in vitro*. Pretreatment with intravenous THC was neuroprotective in rat eyes injected with NMDA and was significantly, but not completely, attenuated with coadministration of the CB_1_ antagonist/inverse agonist SR141716A. These results suggested that CB_1_ is at least partially responsible for the neuroprotective effects seen with this model [69].

Endocannabinoid levels may fluctuate with disease. While decreased endocannabinoid tone has been associated with a number of pathologies, including glaucoma [5], increased endocannabinoid tone has been reported to be neuroprotective [42]. Therefore, strategies that reestablish or increase endocannabinoid levels may provide retinal neuroprotection. Studying the effects of AEA* in vivo* has been particularly difficult due to its instability; therefore, the majority of work that has been performed analyzing the role of AEA in neuroprotection has been either through the use of its stable analogue MetAEA or by decreasing AEA metabolism by inhibition of FAAH [44, 71]. This latter strategy was effective in reducing RGC damage as a result of transient high IOP-induced ischemia whereby RGC loss was attenuated by administration of the FAAH inhibitor URB597. The neuroprotective actions of FAAH inhibition were reported to be due to actions at both CB_1_ and TRPV1 [44]. Interestingly, URB597 was also neuroprotective in a pressure-independent model of RGC loss (axotomy) [71], suggesting that these neuroprotective actions are at least partly independent of IOP modulation. Additionally, Slusar et al. [71] reported in this study that increased RGC survival after axotomy was associated with both CB_1_- and CB_2_-dependent modulation of phagocytic microglia. In another study, MetAEA was also found to significantly recover Thy-1 expression in a model of ischemia-reperfusion. Like previous studies, this effect was blocked with the use of the CB_1_ antagonist SR141716, as well as the TRPV1 antagonist capsazepine [44].

While the role of 2-AG has not yet been extensively studied in models of glaucoma, several studies have found that 2-AG is neuroprotective in other models of neurodegenerative disorders (reviewed in [75]). The actions of 2-AG have been examined in a model of inflammation using lipopolysaccharide (LPS), an inflammation-inducing component of Gram-negative bacteria, in Müller glial cultures. Here, 2-AG reduced proinflammatory cytokines and increased anti-inflammatory cytokines. These actions were thought to be mediated through both CB_1_ and CB_2_, as these effects were significantly diminished by antagonists acting at either of these receptors [22]. The finding that cannabinoids may modulate inflammatory mediators is significant given that several proinflammatory cytokines, such as tumor necrosis factor *α* (TNF*α*), have been reported to play a significant role in glaucomatous RGC death [76–78]. Therefore, reduction of proinflammatory cytokines may represent one mechanism of neuroprotection by cannabinoids.

In addition to reducing proinflammatory cytokines, there is evidence which supports the idea that 2-AG may provide neuroprotection via a CB_1_-dependent reduction of COX-2 expression [79]. Increased COX-2 expression has been implicated as an important contributor to neuronal death in a variety of models, including NMDA-induced excitotoxicity and transient retinal ischemia [45, 72]. Reducing COX-2 upregulation was neuroprotective in these models; however, complete block of COX-2 expression in the eye may not be appropriate in the chronic treatment of glaucoma. In most tissues COX-2 is expressed only through induction; however, in the eye COX-2 is constitutively expressed in some amacrine and ganglion cells [45] as well as in the ciliary body [80], and therefore may have an important role in normal functioning. Additionally, while COX-2 may be upregulated in the retina, as mentioned previously, COX-2 expression was decreased in the ciliary body of human eyes with primary open-angle glaucoma and it is possible that further reduction in these tissues would not be beneficial [40]. Therefore, targeting upstream of COX-2, for example, by altering 2-AG rather than blocking COX-2 itself, may be more appropriate in order to reduce potential side effects.

## 6. Use of Cannabinoids for Long-Term Treatment of Glaucoma

The average glaucoma patient will require treatment over a number of decades; therefore, any drug designed to treat glaucoma will have to be both safe and effective when administered in the long term [6]. Despite the possibilities of formulating cannabinoids for localized ocular delivery, chronic use of conventional cannabinoid ligands poses inherent problems, notably, persistent receptor activation/blockage, tachyphylaxis, and the possibility of unwanted off-target actions and behavioral side effects [4]. Although inhalation of marijuana can lead to transient hyperemia and reduced tear production (reviewed in [1]), so far, most human studies using topical or oral cannabinoids have reported minimal issues [49, 50, 52, 81–83]. One study reported 5 cases of attrition due to corneal irritation; however, 4 of these were given vehicle (mineral oil) [81]. Therefore, it would not appear that ECS-modulating drugs pose any additional considerations for topical delivery compared with current clinical topical treatments [46, 47].

To date, studies evaluating the effectiveness of cannabinoid ligands in long-term use have had variable results. Most studies have reported loss of effect of IOP lowering properties within a matter of hours [49, 50, 57]. However, a study by Hosseini and colleagues [84] found that chronic topical application of 0.5% WIN 55,212-2 was effective at reducing IOP for four weeks. This suggests that certain dosing parameters may allow for better long-term efficacy.

A number of alternative strategies may also aid in circumventing desensitization caused by exogenous cannabinoid administration and are showing significant promise in preclinical studies [71, 75, 85–87]. These include drugs that enhance endogenous endocannabinoid signalling. Endocannabinoids are produced locally on-demand in a stimulus-dependent manner; therefore, modifying their metabolism offers the possibilities of improved target specificity. However, where there has been considerable success with the use of URB597 to increase AEA through FAAH inhibition, this does not necessarily seem to be the case for drugs that inhibit MAG-L. In a study of inflammatory pain, loss of effect through tolerance was an issue with chronic JZL184 administration; however, this was not an issue when the dose was lowered [86]. These studies suggest that increasing 2-AG levels may lead to greater cannabinoid receptor desensitization. Therefore, any drug regimen directed at increasing 2-AG may require careful evaluation in order to identify an appropriate dose and dosing regimen to optimize therapeutic benefit while avoiding receptor desensitization.

Another approach to avoid loss of effect may be with the use of cannabinoid receptor allosteric modulators. Allosteric modulators bind to an alternative (allosteric) binding site distinct from the orthosteric site. Binding of the allosteric modulator induces conformational change in the receptor that affects the affinity and/or efficacy of the main orthosteric ligand (reviewed in [88]). Recently, an allosteric binding site was discovered on CB_1_ [89] and since then a few compounds have been synthesized and used to explore their modulatory potential [90–93]. CB_1_ positive allosteric modulators have the potential to stimulate endocannabinoid-mediated CB_1_ signalling in the absence of any significant CB_1_ desensitization. These agents also lack the behavioral side effects, and hence addictive potential, of cannabinoids that activate CB_1_ [90–93], suggesting that they may be good candidate drugs to explore for the future treatment of glaucoma.

## 7. Conclusion

Increasing evidence suggests that modulation of the endocannabinoid system may show potential for the treatment of glaucoma. Administration of cannabinoids in experimental models can lower IOP and reduce RGC loss, possibly by independent mechanisms. Novel therapeutic strategies, including allosteric modulation and inhibition of endocannabinoid breakdown, may enhance the therapeutic effects seen with direct administration of cannabinoids. However, a better understanding of the components of the ECS, their tissue-specific expression, and the functional role of the ocular ECS is still lacking. This information remains essential in order to move forward with the identification of novel ECS drug targets to prevent retinal neuron loss.

## Figures and Tables

**Table 1:** Studies investigating cannabinoid-mediated neuroprotection in models of glaucoma.

Drug	Delivery	Study	Model	Neuroprotective effect versus vehicle (treatment versus control)
THC	IP	Crandall et al., 2007 [68]	Episcleral vein cauterization	~20–40% increase (10–20% loss)
THC	IV	El-Remessy et al., 2003 [69]	Intravitreal NMDA	~9% of vehicle^*∗*^
CBD	IV	El-Remessy et al., 2003 [69]	Intravitreal NMDA	~4% of vehicle^*∗*^
WIN 55,212-2	Topical	Pinar-Sueiro et al., 2013 [70]	Ischemia-reperfusion (high IOP)	9.88% increase (2.45% loss)
MetAEA	IVit	Nucci et al., 2007 [44]	Ischemia-reperfusion (high IOP)	18.6% increase (9.4% loss)
URB597	IP	Nucci et al., 2007 [44]	Ischemia-reperfusion (high IOP)	15.1% increase (12.9% loss)
URB597	IP	Slusar et al., 2013 [71]	Axotomy	1 week, 19.5% increase (27.9% loss) 2 weeks, 22.7% increase (58.9% loss)
Celecoxib	IP	Sakai et al., 2009 [72]	Ischemia-reperfusion (high IOP)	25.8% increase (39.1% loss)
SC-58236	IP	Ju et al., 2003 [45]	Ischemia-reperfusion (high IOP)	Central, 28.4% increase (27.3% loss) Peripheral, 28% increase (26.8% loss)

IP, intraperitoneal; IV, intravenous; IVit, intravitreal; ^*∗*^study reported quantification of tunnel positive cells only.
